# Crosstalk between Autophagy and Inflammatory Processes in Cancer

**DOI:** 10.3390/life11090903

**Published:** 2021-08-30

**Authors:** Eun-Ji Lee, Hyun-Jeong Kim, Min Sik Choi, Ji-Eun Chang

**Affiliations:** 1 Lab of Pharmaceutics, College of Pharmacy, Dongduk Women’s University, Seoul 02748, Korea; 20161803@dongduk.ac.kr (E.-J.L.); 20161786@dongduk.ac.kr (H.-J.K.); 2 Lab of Pharmacology, College of Pharmacy, Dongduk Women’s University, Seoul 02748, Korea

**Keywords:** autophagy, cancer, inflammation, toll-like receptor, reactive oxygen species, inflammatory cytokine, IκB kinase/nuclear factor-κB, autophagy inhibitors, autophagy activators

## Abstract

Inflammation is an adaptive response to tissue injury, which is a critical process in order to restore tissue functionality and homeostasis. The association between inflammation and cancer has been a topic of interest for many years, not only inflammatory cells themselves but also the chemokines and cytokines they produce, which affect cancer development. Autophagy is an intracellular self-degradative process providing elimination of damaged or dysfunctional organelles under stressful conditions such as nutrient deficiency, hypoxia, or chemotherapy. Interestingly, the signaling pathways that are involved in cancer-associated inflammation may regulate autophagy as well. These are (1) the toll-like receptor (TLR) signaling cascade, (2) the reactive oxygen species (ROS) signaling pathway, (3) the inflammatory cytokine signaling pathway, and (4) the IκB kinase (IKK)/Nuclear factor-κB (NF-κB) signaling axis. Moreover, the studies on the context-specific functions of autophagy during inflammatory responses in cancer will be discussed here. On that basis, we focus on autophagy inhibitors and activators regulating inflammatory process in cancer as useful candidates for enhancing anticancer effects. This review summarizes how the autophagic process regulates these key inflammatory processes and vice versa in various cancers.

## 1. Introduction

Inflammation is an adaptive response to tissue injury caused by infection, wound, or chemical irritation [1]. It is also known to be an essential process for the restoring of tissue functionality and homeostasis [2]. The relationship between inflammation and cancer has emerged as an important topic for numerous researchers for many years. In 1863, Rudolf Virchow suggested that the origin of cancer might be the chronic inflammation sites. This hypothesis was supported by further studies dealing with the association of chronic inflammatory diseases and enhanced cancer risks [3,4,5]. Now it has been established that inflammatory cells, as well as the chemokines and cytokines which they produce, strongly influence tumor development [6]. At the initiation stage of the neoplastic process, inflammatory cells act as effective cancer promoters. As the process progresses, they function to encourage cancer spread and metastasis. On the other hand, the inflammatory cells may also suppress tumor growth.

**Figure 1:**
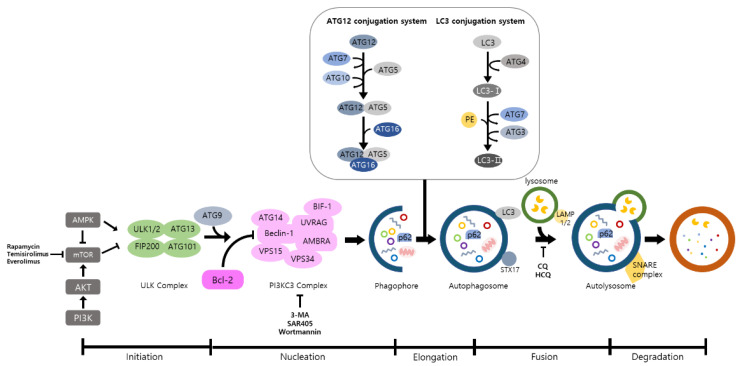
The overall autophagic process and the inhibitors targeting each step. Autophagy includes a multistep procedure: (1) initiation, (2) nucleation, (3) elongation, (4) fusion, and (5) degradation. AMPK and mTOR are major modulators of autophagy and ATGs are deeply associated with this procedure. When the autophagic process is initiated, ULK phosphorylates ATG13 and FIP200 are activated. In nucleation, ULK1 phosphorylates Ambra1, which interacts with Beclin-1, and Beclin-1 forms a PI3KC3 complex with other proteins. After that, cytoplasmic components are enclosed by the phagophore that is expanded to form autophagosomes. These double membranes are fused with lysosomes to form autolysosomes, and various cytoplasmic components are degraded by several enzymes in the autolysosomes. (AMPK: AMP-activated protein kinase; mTOR: mammalian target of rapamycin; ATG: autophagy related gene; ULK: Unc-51 like kinase; FIP200: Focal adhesion kinase family interacting protein of 200-kDa; PI3KC3: Class III phosphatidylinositol 3-kinase).

**Figure 2:**
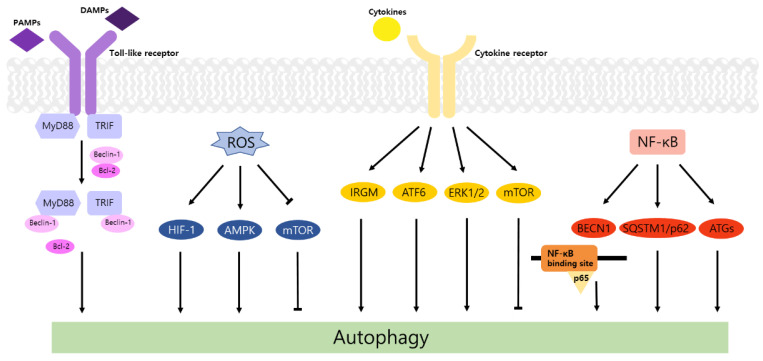
Autophagy signaling pathways involved in cancer-associated inflammation. Four signaling pathways, including (1) toll-like receptor (TLR) signaling cascade, (2) reactive oxygen species (ROS) signaling pathway, (3) inflammatory cytokine signaling pathway, and (4) IκB kinase (IKK)/Nuclear factor-κB (NF-κB) signaling axis, are shown in a schematic illustration. See the text for details. (TLR: toll-like receptor; ROS: reactive oxygen species; IKK: IκB kinase; NF-κB: Nuclear factor-κB).

Autophagy is a well-known intracellular elimination process, which provides degradation of damaged and dysfunctional organelles and proteins under highly-stressed conditions. This term came from the Greek word which represents ‘self-eating’. Figure 1 shows the overall autophagic process and the inhibitors targeting each step. Several studies have shown that close communication between autophagy and apoptosis exists in determining the fate of cells under pathophysiological conditions [7,8]. It is well known that cells use autophagy as a survival mechanism to avoid cell death. However, when the stress increases to a level that exceeds the limits of cellular repair mechanisms, cells inhibit autophagy and initiate the apoptotic cascade [9]. The interaction between autophagy and apoptosis is very complex and entangled with necroptosis; autophagy can function either as an antiapoptotic mechanism or as a proapoptotic mechanism at various stages leading to cell death [10]. Recent research supports the idea that autophagy also plays a pivotal role in regulating inflammation [11]. Various signaling pathways which control tumor-associated inflammation regulate autophagy as well [12]. Inflammatory signals may either induce or inhibit the autophagy process, then autophagy influences tumor-associated inflammation such as induction or inhibition. In addition, autophagy may also regulate key inflammatory cytokines in tumors. Accordingly, autophagy leads to opposing consequences for the tumor since it works as both a tumor suppressor and a tumor promotor [13]. Figure 2 represents autophagy signaling pathways involved in cancer-associated inflammation. In this review, we discuss the relationship between autophagy and the key inflammatory processes in cancer.

## 2. Autophagy and TLR Signaling in Cancer

Toll-like receptors (TLRs) are immune regulators that modulate inflammatory response. They specifically recognize damage-associated molecular patterns (DAMPs) and pathogen-associated molecular patterns (PAMPs) by the innate immune system [14]. Then activated TLRs stimulate diverse transcriptional pathways by the myeloid differentiation primary response gene 88 (MyD88)-dependent or MyD88-independent process. Among them, nuclear factor-κB (NF-κB), mitogen-activated protein kinase (MAPK), and interferon-regulatory factors (IRFs) pathways are associated with the transcription of cancer-related genes that regulate tumor progression, including inflammation, proliferation, angiogenesis, and metastasis. In particular, the NF-κB cascade induces the expression of cytokines, including tumor necrosis factor alpha (TNF-α), interleukin-1 beta (IL-1β), and IL-6, which may cause tumorigenic inflammation [15]. TLRs also regulate the autophagic activity via MyD88- and Toll/IL-1 receptor domain-containing adaptor-inducing interferon-β (TRIF)-dependent pathways. The conjugation of these proteins and Beclin-1 to TLR4 separates the Bcl-2/Beclin-1 complex and triggers the development of autophagosome [14].

In this regard, numerous studies have demonstrated relations between TLR, autophagy, and cancer. One of these studies suggested that TLR4 induces autophagy and performs a protective function against the progression of hepatocellular carcinoma (HCC) [16]. In TLR4 mutant HCC mice models, apoptosis signal-regulating kinase 1 (ASK1)/p38 MAPK/NF-κB and IRF3/IFN pathways were inhibited, and DNA repair protein Ku70 and inflammatory cytokines such as IL-1β, IL-6, IL-12, TNF-α, and IFN-γ were reduced due to the damaged immune system. In addition, the levels of reactive oxygen species (ROS) and p62 were increased while the expression of light chain 3 (LC3)-I/II, Beclin-1 and class III phosphatidylinositol 3-kinase (PI3KC3) were decreased. In terms of tumorigenesis, TLR4 mutant mice showed an accelerated progression of HCC and shortened survival time compared to wild-type HCC mice. In brief, cancer development may be delayed by mitigating genetic instability due to impairment of DNA repair, and this process is closely connected with TLRs and autophagy. 

In contrast, TLR4 enhances autophagy induction through beclin 1 (BECN1) ubiquitination by TNF receptor-associated factor 6 (TRAF6) promoting cell migration and invasion in various cancer cell lines [17]. Elevation of LC3-II levels and reduction in cell migration/invasion were detected in additional treatment with chloroquine (CQ), the lysosomal inhibitor. Moreover, the expression of IL-6, matrix metalloproteinase 2 (MMP2), and CC chemokine ligand 2 (CCL2) was augmented in A549 cells and was significantly higher in p62KO A549. These results indicate that TLR4 induces autophagy and cell migration/invasion, which is negatively controlled by p62.

Similarly, TLR9 induces autophagy and stimulates cell proliferation and invasion of HCC. TLR9, ATG, LC3B, and BECN1 were known to be expressed in human HCC cell line, Huh7. Additionally, epithelial mesenchymal transition (EMT) bioindicators, including vimentin and snail showed an increasing pattern as the TLR9 upregulated. Hydroxychloroquine (HCQ) is a suppressor of TLR9 and autophagy. Therefore, HCQ inhibits formation of autolysosome and affects progression of HCC. After treating with HCQ, TLR9 and EMT bioindicators were reduced, and cell viability and migration/invasion were also inhibited [18].

Taken together, TLRs stimulate the activation of autophagy and play a dual role in tumorigenesis. Different outcomes in tumor development are based on the type of TLR or cancer.

## 3. Regulation of Autophagy by ROS in Inflammation and Cancer

ROS is the highly reactive byproduct, which is generated by oxidative phosphorylation in the metabolic process. It is mainly divided into free radical oxygen and nonradical oxygen [19]. In most cases, ROS is generated endogenously by peroxisome and mitochondria. Mitochondrial ROS participates in the normal metabolic pathway; however, excessive ROS may cause oxidative stress and metabolic diseases, leading to cell death and inflammation. ROS has a crucial role in mediating inflammation. Once inflammation occurs, phagocytosis of neutrophils is activated and ROS is increased at the site of infection. ROS regulates the NLR family pyrin domain containing 3 (NLRP3) inflammasome. Thioredoxin-interacting protein (TxNIP) represses thioredoxin (Trx) to induce ROS and increases activity of the inflammasome. Moreover, TxNIP is separated from Trx and interacts with NLRP3 inflammasome [20]. It may transfer pro IL-1β, pro IL-18 to IL-1β, and IL-18 via active caspase-1 and induce inflammatory response [21].

According to previous studies, ROS is known to stimulate autophagy. In breast and ovarian cancers, ROS enhances activity of autophagy and improves cancer cell survival [22]. Generated ROS induced autophagy by LC3 accumulation and increased the Beclin-1/Bcl-2 ratio [23]. In a recent study, when the ROS level is increased in prostate cancer, acidic vesicular organelles (AVO) and conversion of LC3-I to LC3-II hallmarks of autophagy are triggered [24]. In an oxygen and nutrient deficient environment, excess ROS increases the ratio of AMP/ATP, activates AMP-activated protein kinase (AMPK) [25], and induces formation of autophagosome via activation of UNC-51-like kinase (ULK) and phosphorylation of Beclin-1 [26]. ROS also induces autophagy by repressing the PI3K-Akt-mTOR pathway [27]. When cancer cells are under starvation conditions, H_2_O_2_ is generated, and cysteine protease ATG4 is restrained. This process elevates the formation of autophagy [28]. Recent studies demonstrated that the ROS level is increased in cancer cells and it stimulates autophagy. In colorectal and gastric cancers, beclin-1, the autophagy-related gene is highly expressed [29]. Autophagy impedes DNA mutations by preventing ROS accumulation through elimination of damaged mitochondria. Therefore, inhibition of autophagy via loss of Atg5 and Atg7 helps to initiate tumors by inducing accumulation of ROS and inflammation which is related to IL-1β, IL-18 [30].

Cancer cells show a high correlation with hypoxia due to their active metabolism. The translational factor, hypoxia-inducible factor (HIF), is the major mediator of hypoxia. The HIF-1 pathway encourages angiogenesis and provide oxygen to oxygen-deficient tissue [31]. In hypoxia, ROS is generated, and stabilized HIF-1α interacts with HIF-1β and binds to specific DNA sequence to make epigenetic changes [32]. This stimulates autophagy and promotes survival of cancer cells. Moreover, ROS upregulates cyclin mRNA which progresses the cell cycle from the G1 phase to the S phase. A low ROS level decreases cell proliferation and induces apoptosis since G1/S transition is restricted [33]. An excess of ROS, especially H_2_O_2_, reduces activity of MAP kinase phosphatase 3 (MKP3) and activates extracellular signal-regulated kinase (Erk)1/2 [34] to promote cell survival and adhesion-independent cell growth [35]. 

NADPH oxidase 2 induces ROS production and deactivates AMPK increasing colon cancer metastasis [36]. In addition, other important sources of ROS generation such as mitochondrial electron transport chain complexes [37], xanthine oxidase [38], and cyclooxygenase-2 [39] also play critical roles in the regulation of autophagy and the inflammatory process in cancer.

Taken together, ROS correlates with the whole inflammation process. Inflammatory factors influence activation of autophagy. ROS production is related to massive metabolic process in cancers and this leads to the autophagy process. 

## 4. Autophagy and Inflammatory Cytokines in Cancer

Cytokines, products of immune and other cells, bind to specific receptors leading to a pleiotropic activity. This activity induces distinct effects of cytokines on different cells [40]. Moreover, cytokines act as a crucial mediator in inflammatory responses [40] that are augmented by some proinflammatory cytokines, such as IFN-γ [41], TNF-α [42], IL-1β [43], IL-6 [44], and IL-8 [45]. The interaction between cytokines and autophagy may lead to various results in tumor development. According to previous studies, inflammatory cytokines may either activate or inactivate autophagy in cancer.

IFN-γ induces autophagy and inhibits tumor growth in the human lung epithelial cancer cell line, A549. p62 degradation and LC3 conversion indicating the formation of autophagosome were observed in A549 cells treated with IFN-γ. Additional treatment with CQ caused the accumulation of p62 and LC3-II. By silencing immunity-related GTPase family M protein (IRGM) and activating transcription factor 6 (ATF6) via shRNA, it was confirmed that IRGM- and ATF6-related signaling pathways were associated with autophagy induced by IFN-γ. Moreover, IFN-γ signaling not only impeded cell proliferation but also played cytotoxic roles including stimulation of apoptotic or nonapoptotic cell death. In shAtg5, lactate dehydrogenase (LDH) activity, which means cytotoxicity, was attenuated compared to shLuc as a negative control [41].

TNF-α has shown inconsistent effects on autophagic activity. In the breast cancer cell line, MCF-7, TNF-α triggered autophagy via the ERK1/2 signaling pathway [46]. On the other hand, TNF-α inhibited autophagic activity induced by trovafloxacin (TVX) in the HepG2 human HCC cell line [42]. TVX, a hepatoxic drug, induced autophagy by suppressing the mammalian target of rapamycin (mTOR) pathway, leading to protective roles against cytotoxicity. TNF-α prompted mTOR pathway through phosphorylation of p70S6K, which led to inhibition of autophagy. Thus, LC3-II and GFP-LC3 puncta were attenuated, and cell viability was reduced in TVX-treated HepG2 cells after treatment with TNF-α. 

IL-6 alleviates the induction of autophagy during tumor progression. In ovarian cancer cell NIH-OVCAR3, IL-6 blocked the accumulation of autophagosome and GFP-LC3 puncta at the area, which is deeply involved in migration activity. Furthermore, it was observed that cell migration was suppressed by IL-6 stimulation. These findings are associated with the expression of ARH-1 and AKT/mTOR/signal transducer and activator of transcription 3 (STAT3) pathway. ARH-1 acts as an autophagy inducer combining with Beclin-1 and activating the PI3KC3 complex. Data showed that IL-6 decreased the expression of ARH-1 and stimulated the phosphorylation of AKT, mTOR, and STAT3 and in turn, led to cell invasion [44].

Similarly, IL-8 secreted by cancer-associated fibroblasts (CAFs) also reduces autophagy and stimulates cell migration of ovarian cancer [45]. CAFs, major components of stromal cells, are included in the tumor microenvironment (TME) with immune cells and the extracellular matrix (ECM). The interplay between the tumor cell and TME, which is closely related to cytokines and autophagy, influences tumorigenesis [47]. Ovarian cancer-associated fibroblasts (OVCAFs) secreted a greater amount of IL-8 than ovarian normal-associated fibroblasts (OVNFs). Due to enhanced IL-8, LC3-II/LC3-I decreased, and p62 accumulation increased in human ovarian cancer cell lines. Moreover, cell migration was promoted in a wound healing assay. 

Autophagy also regulates the level of cytokines in several cancers. Induced autophagy in CAFs can stimulate secretion of IL-6 and IL-8 followed by aggressive tumor development. In head and neck squamous cell carcinoma (HNSCC) cell lines, CAFs showed a potent autophagic activity compared to normal fibroblast (NF) and an increase in LC3-II under CQ treatment. Moreover, the outcomes of this study displayed a decrease in the density of IL-6 and IL-8 through knockdown of Beclin-1 and a reduction in cell migration and invasion of the tumor in siBECN, siATG7, or CQ-treated CAF-CM. The addition of IL-6 and IL-8 to siBECN promoted cell migration. Importantly, autophagy and cytokines such as IL-6 and IL-8 form a feedback loop during this process. IL-6 and IL-8 secreted by CAFs or HNSCC cells may stimulate CAF autophagy [48]. 

Pancreatic stellate cells (PSCs), which are part of stromal cells in TME, regulate tumor deterioration by interacting with pancreatic cancer cells (PCCs). Induced autophagy in PSCs elicits a change from inactive PSCs to active PSCs, which release cytokines and ECM. These released factors are associated with the invasion and migration of PCCs. Based on experimental data, LC3 expression was confirmed in activated PSCs. In CQ-treated PSCs, reduced autophagy resulted in the accumulation of LC3-II and p62. By knocking down Atg7 to inhibit the autophagy, the production of IL-6 decreased in PSCs, and the migration and invasion of PCCs were delayed [49]. As mentioned above, autophagy may prompt the secretion of IL-6. However, another study suggests that autophagy may reduce the IL-6. In 66cl4 breast cancer cell lines from mice, which spread to the lung, autophagic activity was substantially intensified, and LC3-II was increased in CQ- treated 66cl4 cells. Moreover, an increase in IL-6 and decrease of cell viability were observed upon treatment with CQ [50].

In summary, cytokines and autophagy represent protumor or antitumor effects under the unclear roles for each other, and the mechanisms have not been completely understood. The results discussed above showed the relation between the duality of autophagy in tumorigenesis and cytokines. Generally, it is known that IFN-γ, TNF-α, and IL-6 promote autophagy while IL-10 inhibits autophagy via diverse signaling cascades [51]. However, in this review we suggested that TNF-α and IL-6 may both promote and inhibit autophagy in certain cases. The action of autophagy on cytokine secretion has also been suggested. Autophagy increases the production of IL-6 and TNF-α in diverse cell types, which induces innate immune responses [51], and similar results were observed in cancer. Conversely, some studies showed that autophagy inhibited the expression of IL-6 [50] and TNF-α [52], and further studies are required to determine the relationship with tumorigenesis.

## 5. Interaction between IKK/NF-κB Axis and Autophagy in Cancer

As mentioned above, it can be seen that NF-κB is a key molecule involved in both TLR-, ROS-, and cytokine-related mechanisms interacting with autophagy in the inflammatory process in cancer. The correlation between NF-κB itself and cancer has been elucidated [53]. The key point is that NF-κB regulates tumor-promoting inflammation or antitumor immune processes through crosstalk with signaling mechanisms related to proteins such as STAT3, p53, and mTOR [54,55,56]. In this section, among these regulatory mechanisms, we would like to review the relationship between the autophagy process and the NF-κB pathway in cancer. 

In promoting or limiting tumorigenesis, autophagy and NF-κB regulate each other’s activity through complex interactions [57,58]. Among the various experimental results related to these interactions, the following three potential mechanisms have been suggested.

First, NF-κB enhances autophagy by inducing the expression of BECN1, SQSTM1/p62, and other autophagy-related proteins. A conserved NF-κB binding site in the promoter region of the human and murine BECN1 gene is also known as Atg6 [59]. As this binding site in the BECN1 promoter region interacts with p65, induction of p65 by stimuli involving inflammatory processes is linked to BECN1 upregulation and the following autophagy induction. In another study, it has been reported that NF-κB positively regulates p62 expression [60]. The results have shown that chloroquine, an autophagy inhibitor, increased the p62 protein level not only by increasing p62 stability but also by increasing p62 expression. Using NF-κB inhibitors and RELA knockdown, it has been found that NF-κB is required for chloroquine-induced expression and upregulation of p62.

Second, autophagy controls the IκB kinase (IKK)-NF-κB signaling axis and vice versa [57,58]. Hsp90, a molecular chaperone, is a key stability factor for IKK signal activation [61]. It has been known that acute stress can induce the dissociation of Hsp90 from the IKK complex, which results in the inhibition of NF-κB signaling [62]. In this regard, it has been demonstrated that geldanamycin, an Hsp90 inhibitor, increased the degradation of IKK (α and β) through autophagy. Moreover, it was confirmed that ubiquitination and proteasomal degradation were not involved in this IKK degradation [63]. In contrast, it has been demonstrated that starvation stimulated Atg5, BECN1, and LC3 expression in the IKK-dependent but NF-κB-independent manner [64]. These results suggest that IKK can induce autophagy through the expression of autophagy related genes (ATGs) without the involvement of NF-κB activation. In addition, it has been reported that several autophagy inducers increased autophagic flux through IKK complex activation [65]. 

Thirdly, contrary to the above, there have been several reports in which NF-κB signaling acts negatively on the autophagic process. These cases can be broadly divided into the following two categories. One is that NF-κB signaling activation stimulates the expression of autophagy repressor proteins, such as Bcl-2 and Bcl-xL [66,67]. The other is that NF-κB signaling activation suppresses the expression of autophagy inducer proteins, including JNK1 and BCL2 interacting protein 3 (BNIP3) [68,69].

**Figure 3:**
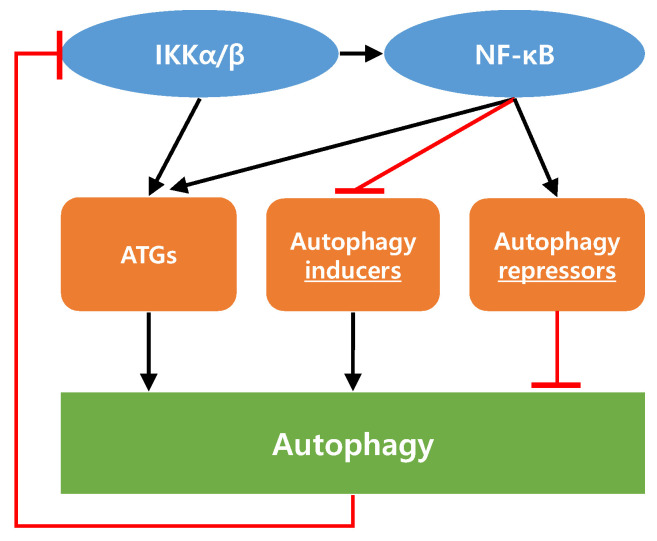
Interaction between autophagy and the IKK–NF-κB axis. Both IKK and NF-κB can enhance autophagy by inducing the expression of autophagy-related proteins. Increased autophagic flux can inhibit IKK signaling by degrading IKK components. NF-κB signaling can act negatively on the autophagic process through the increase in autophagy repressors or through the decrease in autophagy inducers. Refer to the main text for proteins in each category in detail.

The above studies demonstrate that the NF-κB signaling pathway can control autophagy and vice versa. Considering that NF-κB acts as a key regulator of the inflammatory process, the interaction between NF-κB signaling and the autophagic process in cancer should be clarified. The interaction between autophagy and the IKK–NF-κB axis is described in Figure 3. Table 1 summarizes the signaling pathways and autophagy in various cancer types.

## 6. Autophagy Inhibitors and Activators for Cancer Treatment 

The role of autophagy in cancer still remains controversial. Autophagy may possess dual roles in cancer development as inhibitors and activators. Inhibition of autophagy may induce sensitized cancer cells leading to enhanced antitumor efficacy over standard cancer treatments such as chemotherapy and radiotherapy. On the other hand, activation of autophagy increases tumor cell death from apoptosis-resistant cells since excessive autophagy plays a critical role in the pro-death pathway. Numerous studies evaluating the anti-tumor effect of using autophagy inhibitors and activators are already completed or ongoing. In this section, we focus on autophagy inhibitors and activators, which influence inflammation therefore enhancing anticancer effects.

### 6.1. Autophagy Inhibitors for Cancer Treatment

3-Methyladenine (3-MA) is one of the well-known autophagy specific inhibitors. It was first discovered by Seglen et al. in 1982 [70]. Phosphoinositide 3-kinase (PI3K) and Vps34 are the molecular targets for 3-MA which regulate autophagy process. Inhibiting autophagy with 3-MA in cancer may stimulate ROS formation and treatment with cytotoxic agent increases ROS accumulation. In this manner, Kaminskyy et al. suggested that blocking autophagy leads to reduced lung cancer cell proliferation and enhanced apoptosis [71]. Interestingly, Yoon et al. discovered that blocking autophagy with 3-MA in HeLa cells decreases ROS generation leading to inactivation of proinflammatory STAT3 and IL6 [72]. Finally, cancer cells showed poor survival despite an opposite ROS expression level. From diverse previous studies, pretreatment of 3-MA combined with other anti-cancer agents showed improved antitumor efficacy in various cancer types [73,74,75]. In addition, Chen et al. reported that inhibition of autophagy with 3-MA in esophageal cancer contributed to radiation sensitization [76]. In contrast, when autophagy was downregulated with 3-MA in melanoma, crosspresentation of tumor antigens were abolished [77]. This led to enhanced tumorigenesis, which revealed the opposite result against aforementioned studies. SAR405 and wortmannin are also PI3K targeted autophagy inhibitors. Combining SAR405 with an mTOR inhibitor, everolimus in renal cancer showed reduced cell proliferation [78]. In addition, inhibited autophagy with wortmannin enhanced the antitumor effect in HeLa cells [79].

### 6.2. Autophagy Activators for Cancer Treatment

Rapamycin is a representative autophagy activator, which is categorized as a mTOR inhibitor. It is produced by Streptomyces hygroscopicus exhibiting antifugal, immunosuppressive, and anti-cancer activities [80]. The mTOR signaling pathway takes part in tumor growth and proliferation. From Zhao et al.’s study, when autophagy was activated with rapamycin in ovarian cancer, intracellular ROS levels were decreased, and migration and invasion were inhibited through reversing EMT. Furthermore, expression of Zeb1 was decreased [81]. EMT plays a crucial role in tumor spreading, which leads to metastasis [82]. Zeb1 is also known to be related to metastasis of various cancer types [83,84]. In another study from Yan’s group, autophagy was induced with rapamycin treatment in a melanoma metastasis mouse model. STAT1 activity was enhanced with increased antimetastatic effect. In this study, triggering TLR4 and TLR9 showed synergetic antitumor efficacy with rapamycin induced autophagy; therefore, a combination therapy of immunotherapy and autophagy activator was suggested to be a novel therapeutic approach against tumor progression and metastasis. Rapamycin was also reported to be effective in many different cancers [85,86]. Temsirolimus and everolimus are rapamycin analogs, which improved the water solubility and stability of rapamycin. Temsirolimus is proved to be effective in the treatment of prostate cancer [87], colorectal cancer [88], and advanced solid tumors [89]. Everolimus has also been investigated in various tumor types such as triple-negative breast cancer [90], renal cell carcinoma [91], and advanced solid tumors [92].

## 7. Conclusions

In summary, we reviewed how inflammation-related signaling regulates to the autophagy process and, conversely, how autophagy controls inflammation-mediated signaling in cancer. We investigated how autophagy may exhibit a variety of functions in inflammation-based signaling that can influence cancer initiation and progression in various ways. As the results on the context-specific functions of autophagy accumulate, its position as a specialized target according to the type or characteristics of cancer is becoming more solid. Despite the role of autophagy in cancer still remaining controversial, elucidating more specific interactions between autophagy and the inflammatory process may provide important clues.

## Figures and Tables

**Table 1:** Signaling Pathways and Autophagy in Various Cancer Types.

Cancer Types	Signaling Pathways	Autophagy	Therapeutic Effects	Refs.
Hepatocellular Carcinoma	TLR4	↑	Positive	[16]
	TLR9	↑	Negative	[18]
	TNF-α	↓	Positive	[42]
Colorectal cancer	ROS	↑	Positive	[23]
Prostate Cancer	ROS	↑	Positive	[24]
Lung Cancer	IFN-γ	↑	Positive	[41]
Breast Cancer	TNF-α	↑	Negative	[46]
	IL-6	↓	Positive	[50]
Ovarian Cancer	IL-6	↓	Negative	[44]
	IL-8	↓	Negative	[45]
Head and Neck Squamous Cell Carcinoma	IL-6, IL-8	↑	Negative	[48]
Pancreatic Cancer	IL-6	↑	Positive	[49]

↑: Autophagy induction, ↓: Autophagy inhibition.

## Abbreviations

**TLR**	**Toll-Like Receptor**
DAMP	damage-associated molecular pattern
PAMP	pathogen-associated molecular pattern
MyD88	myeloid differentiation primary response gene 88
NF-κB	nuclear factor-κB
MAPK	mitogen-activated protein kinase
IRF	interferon-regulatory factor
TNF-α	tumor necrosis factor- α
IL-1β	interleukin-1β
TRIF	Toll/IL-1 receptor domain-containing adaptor-inducing interferon-β
ASK1	apoptosis signal-regulating kinase 1
ROS	reactive oxygen species
LC3	light chain 3
PI3KC3	class III phosphatidylinositol 3-kinase
BECN1	beclin 1
TRAF6	TNF receptor-associated factor 6
CQ	chloroquine
MMP2	matrix metalloproteinase 2
CCL2	CC chemokine ligand 2
EMT	epithelial mesenchymal transition
HCQ	hydroxychloroquine
AMPK	AMP-activated protein kinase
HIF	hypoxia-inducible factor
IRGM	immunity-related GTPase family M protein
ATF6	activating transcription factor 6
ERK	extracellular signal-regulated kinase
TVX	trovafloxacin
STAT3	signal transducer and activator of transcription 3
CAF	cancer-associated fibroblast
TME	tumor microenvironment
ECM	extracellular matrix
IKK	IκB kinase
PI3K	phosphoinositide 3-kinase
ATG	autophagy related gene
3-MA	3-Methyladenine
